# Supplementary material for: MIMIC: a flexible pipeline to register and summarize IMC-MSI experiments
Source: bioRxiv. 2025 Jul 11:2025.07.08.663623. Preprint. [Version 1] doi: 10.1101/2025.07.08.663623 (PMC12265742; doi:10.1101/2025.07.08.663623)
Supplement: Supplementary file 1 [file media-1.pdf]

MIMIC: a flexible pipeline to register and summarize IMC-MSI experiments
Supplementary Figures

Reto Gerber

July 8, 2025

1 Supplementary

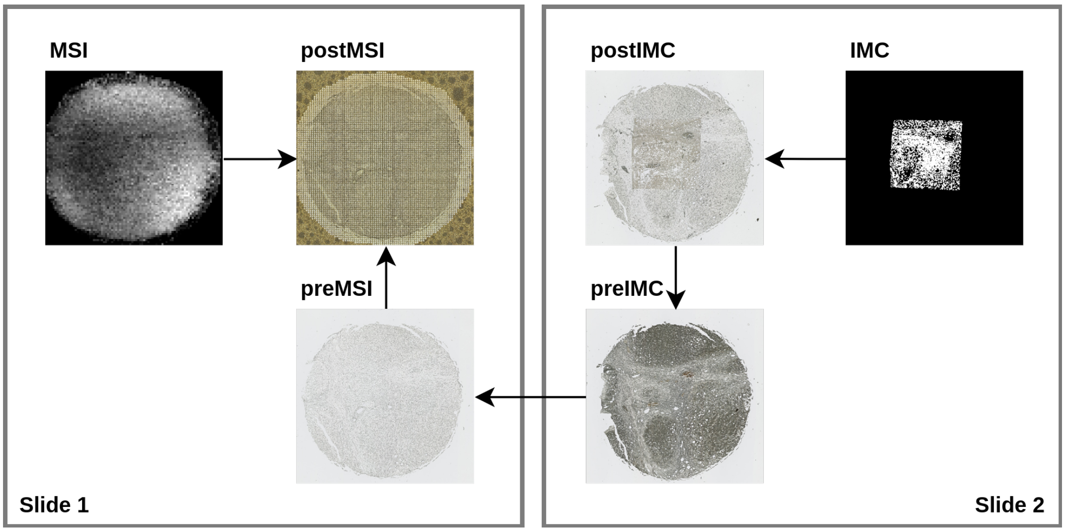

Figure 1: Broad overview of registration workflow with example images.

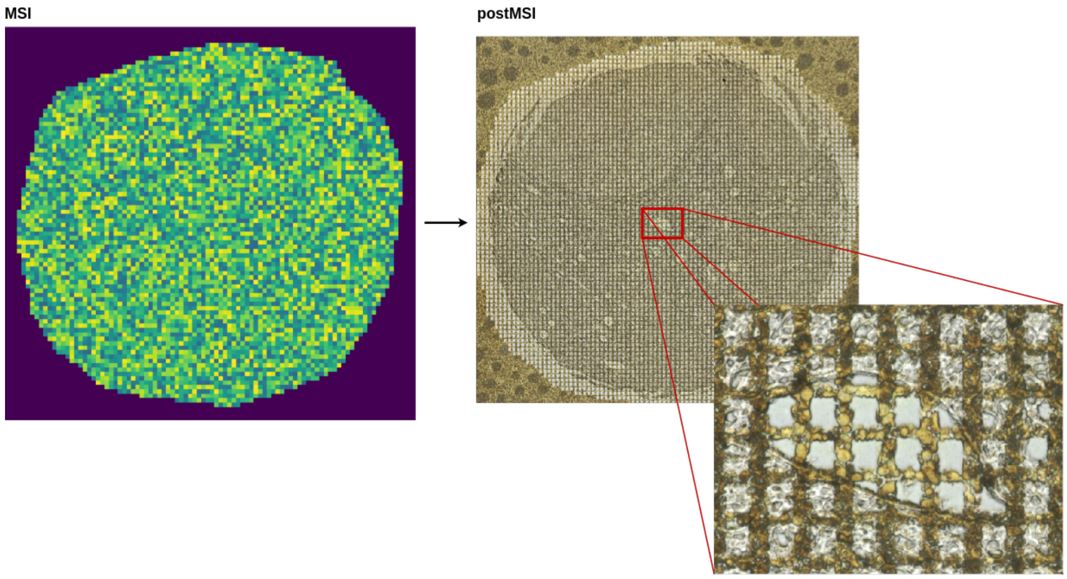

Figure 2: Example MSI to postMSI registration.

**Registration Step**	**Source Image**	**Target Image**	**Type of Comparison**	**Metric**	**Source Feature**	**Target Feature**
MSI to postMSI	MSI	postMSI	landmark	MLD	pixel coordinate	ablation mark coordinate
IMC to postIMC	IMC	postIMC	landmark	MLD	grid of coordinate points after transform	grid of coordinate points before transform
postIMC to preIMC	postIMC	preIMC	landmark	MLD	coordinate of RoMa feature	coordinate of RoMa feature
postIMC to preIMC	postIMC	preIMC	region	DICE	region mask from SAM	region mask from SAM
preIMC to preMSI	preIMC	preMSI	landmark	MLD	coordinate of RoMa feature	coordinate of RoMa feature
preIMC to preMSI	preIMC	preMSI	region	DICE	region mask from SAM	region mask from SAM
preMSI to postMSI	preMSI	postMSI	landmark	MLD	coordinate of RoMa feature	coordinate of RoMa feature
preMSI to postMSI	preMSI	postMSI	region	DICE	region mask from SAM	region mask from SAM

Table 1: Registration steps and their corresponding precision evaluations. MLD: Median landmark distance.

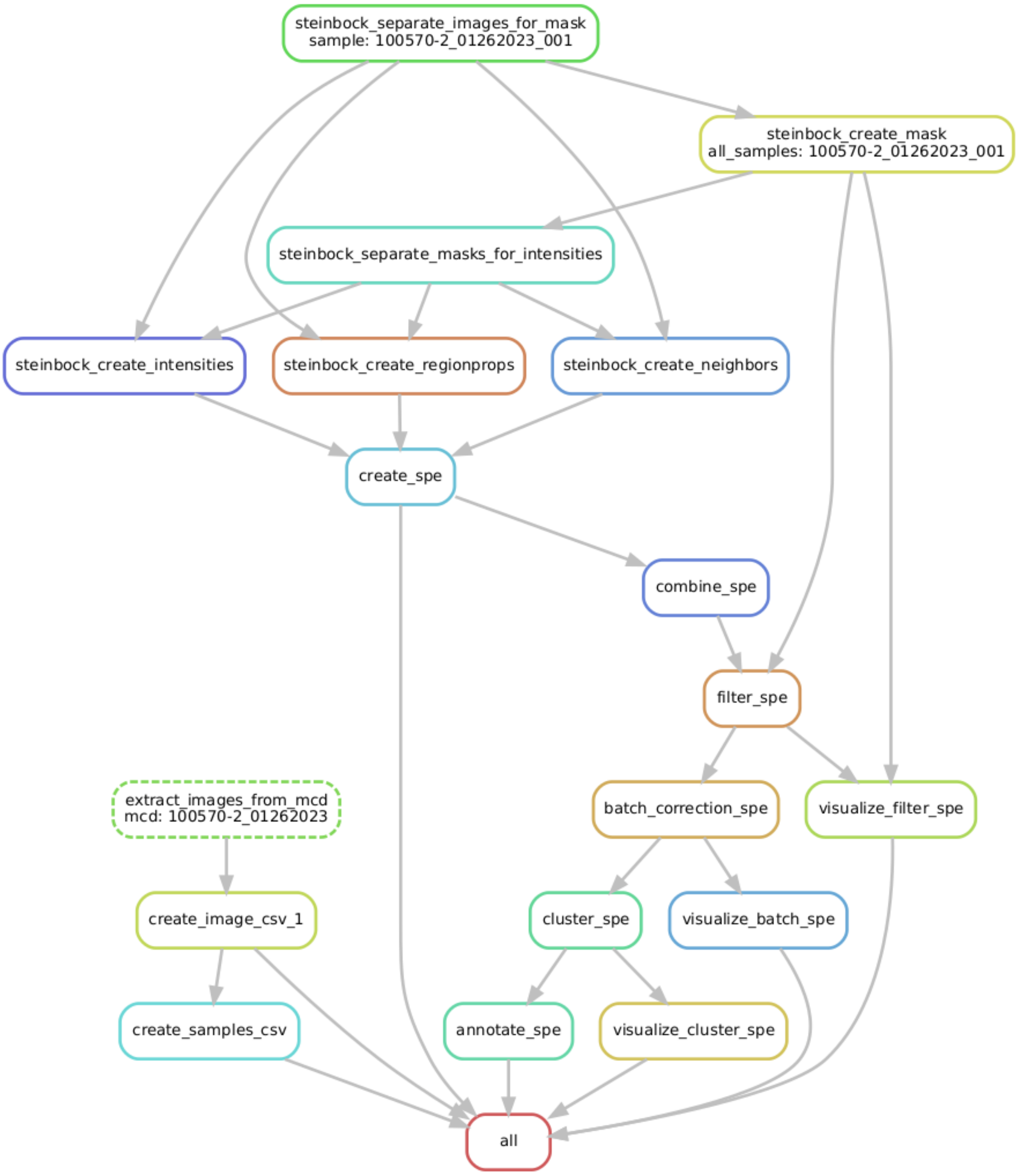

Figure 3: DAG of Snakemake workflow for IMC processing.

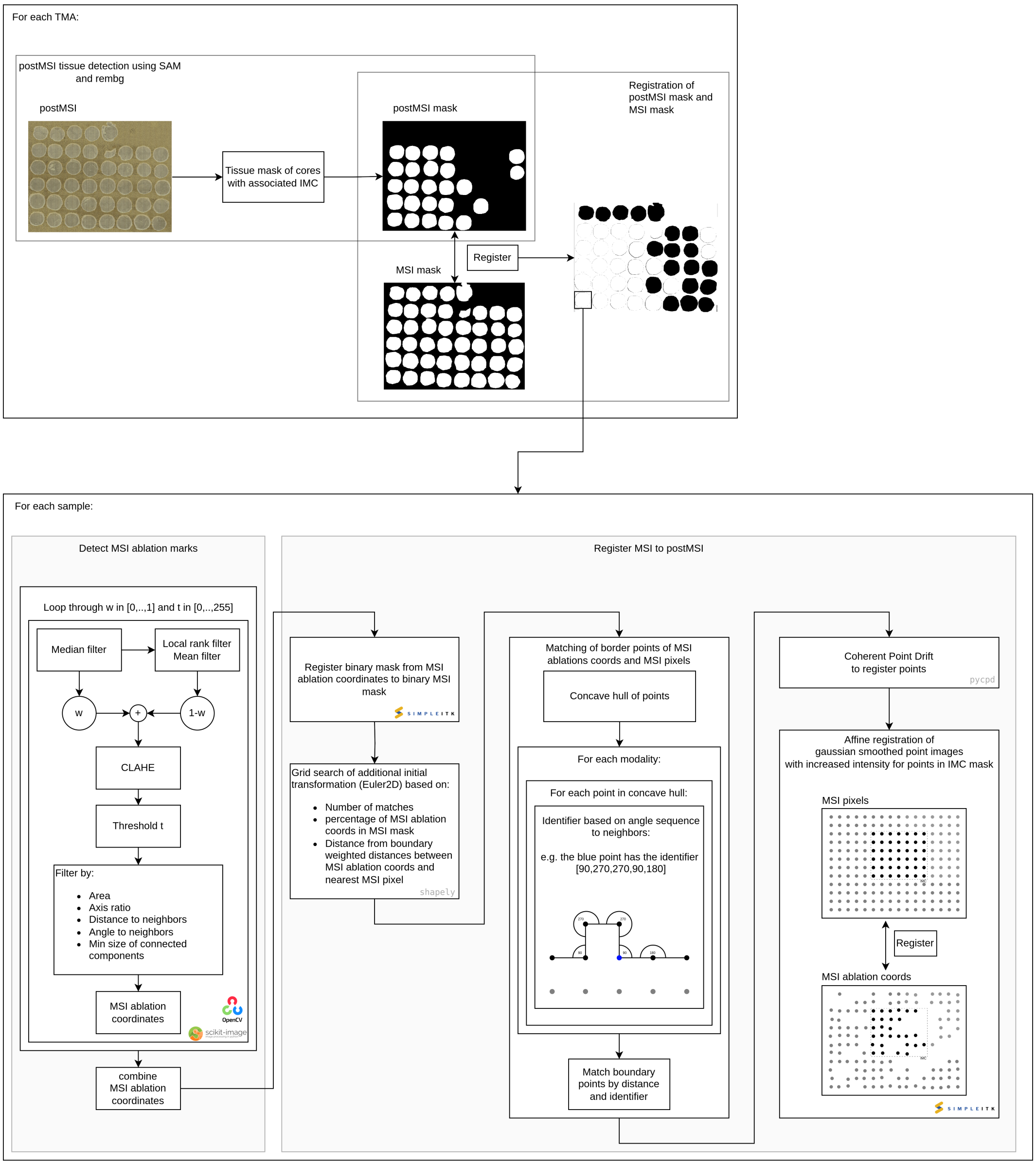

Figure 4: Diagram of the automatic registration of MSI to postMSI. First the TMA cores from the postMSI image and the MSI are matched (top panel). Next for each TMA core registration of MSI to postMSI ablation marks is done (bottom panels). After detection of the MSI ablation marks (bottom left panel) the detected marks are coregistered with the MSI pixels (bottom right panel).

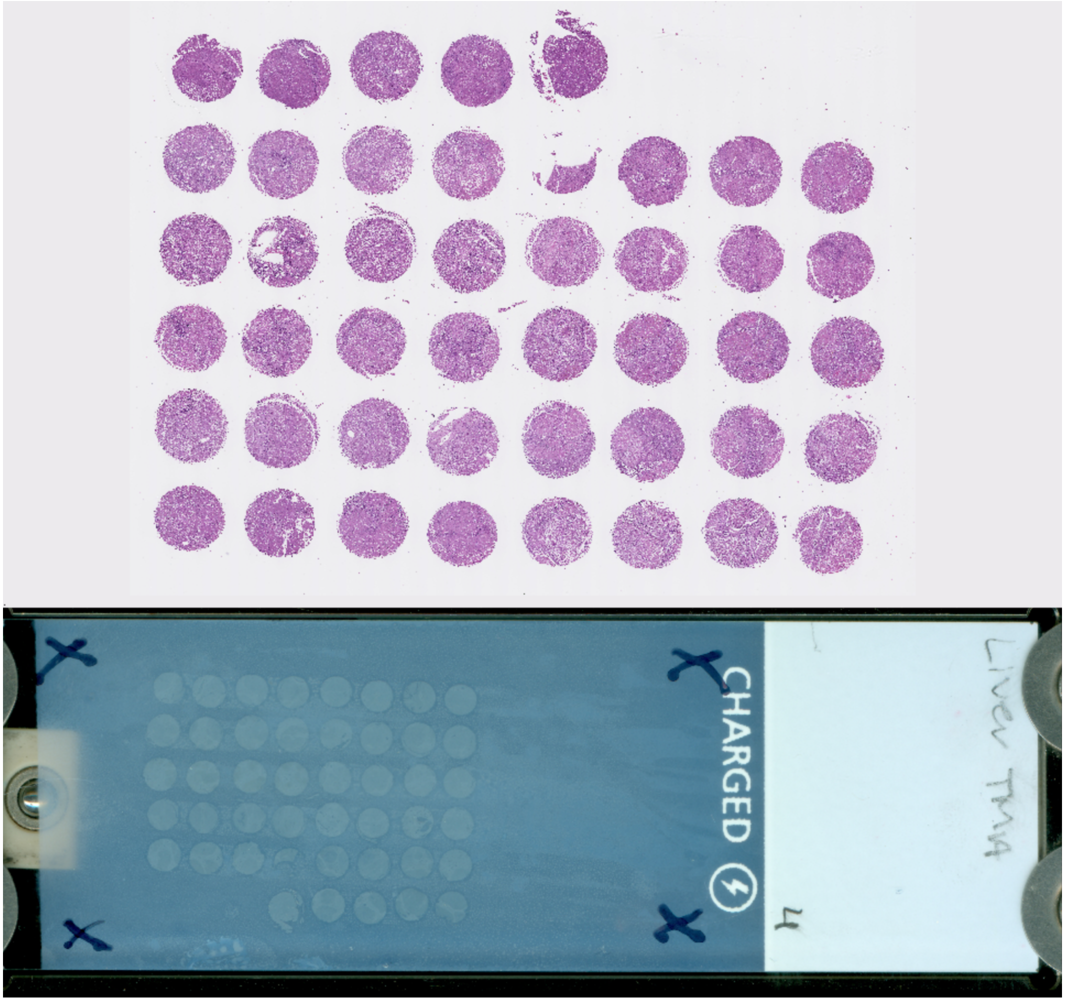

Figure 5: Images used for manual vs automatic image registration precision. Top: high resolution H&E stain, bottom: low resolution slide scan.

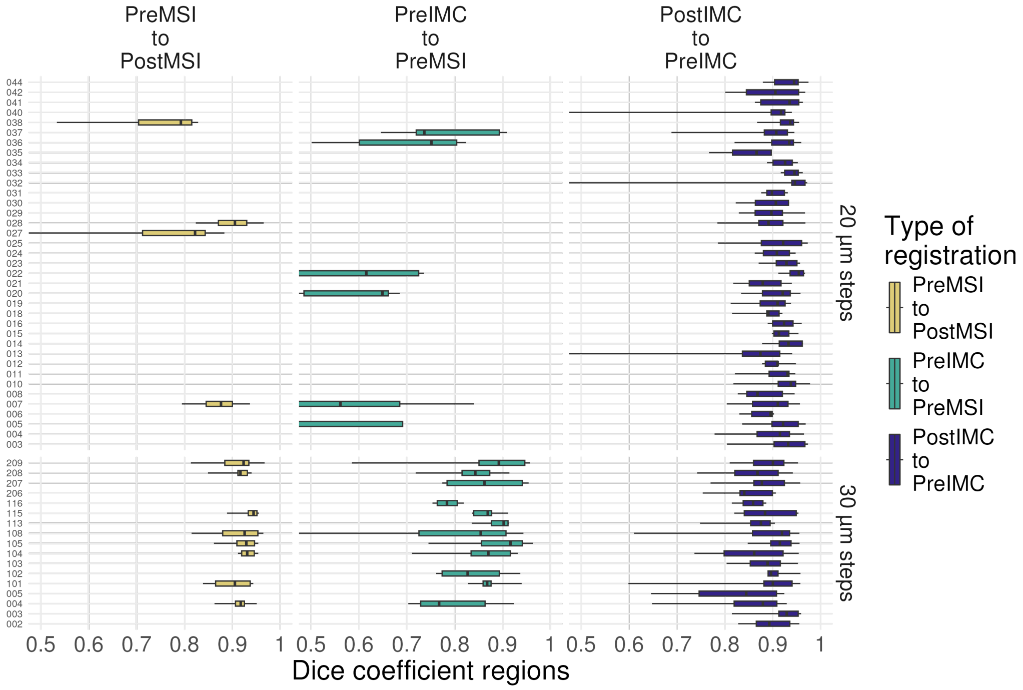

Figure 6: Evaluation precision of registrations. DICE coefficients of regions extracted from the four microscopy images and compared agains each other.

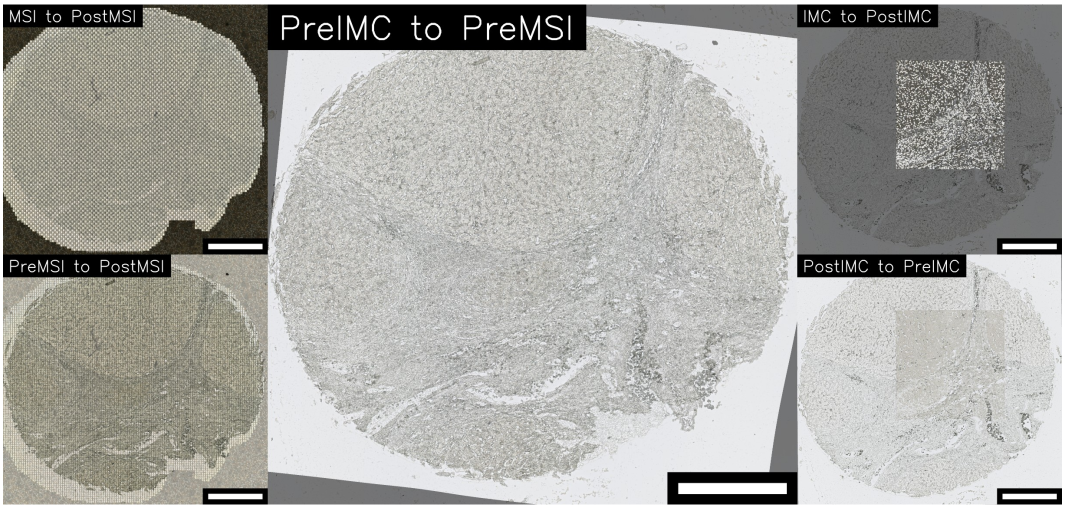

Figure 7: Blended images after co-registration for sample NASH 005.

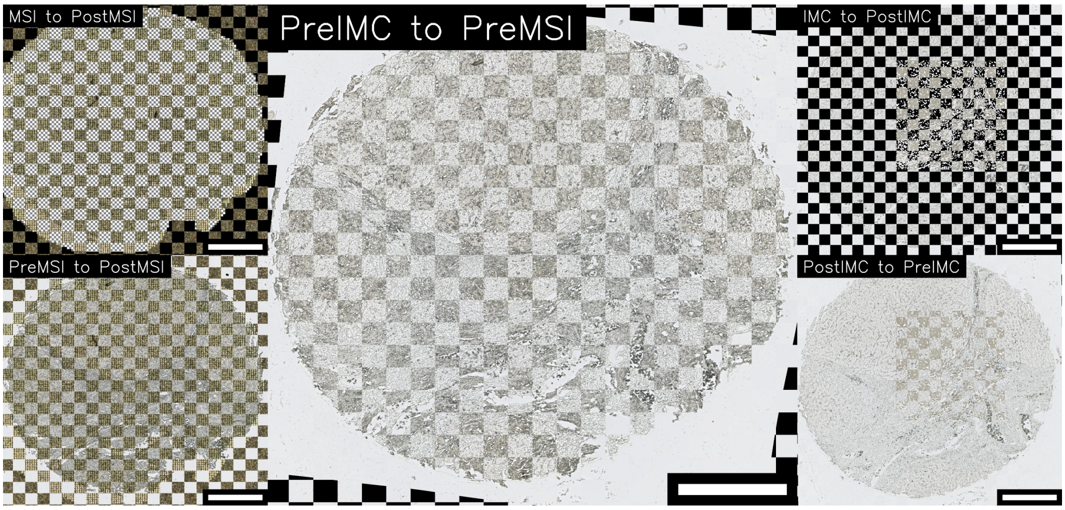

Figure 8: Checkerboard images after co-registration for sample NASH 005.

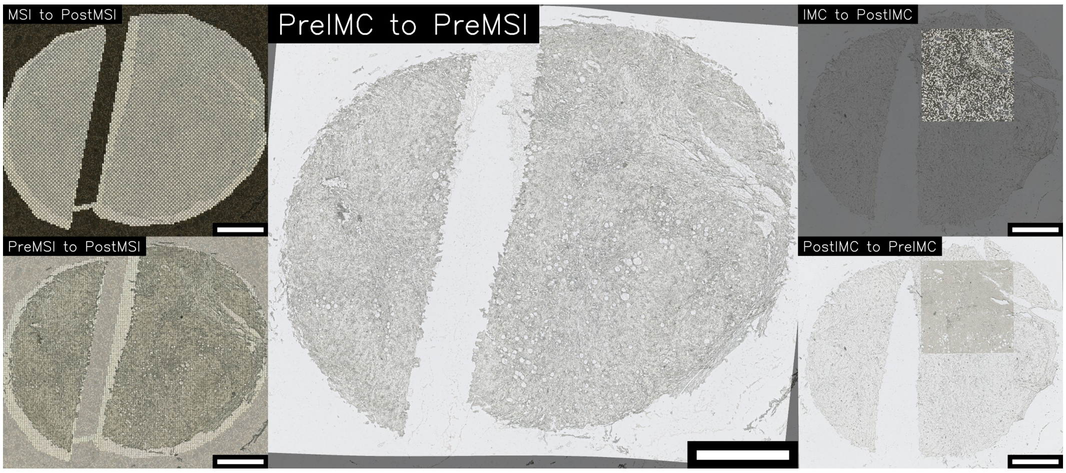

Figure 9: Blended images after co-registration for sample NASH 011.

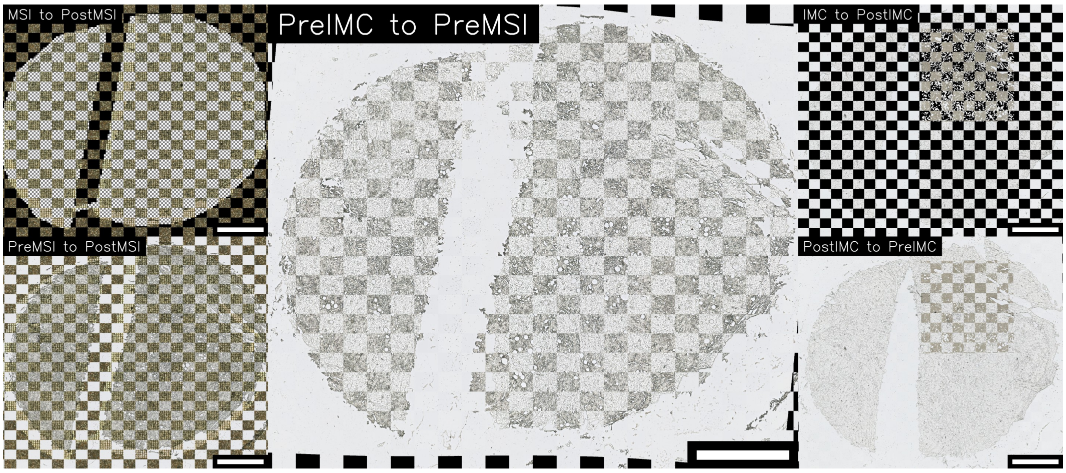

Figure 10: Checkerboard images after co-registration for sample NASH 011.

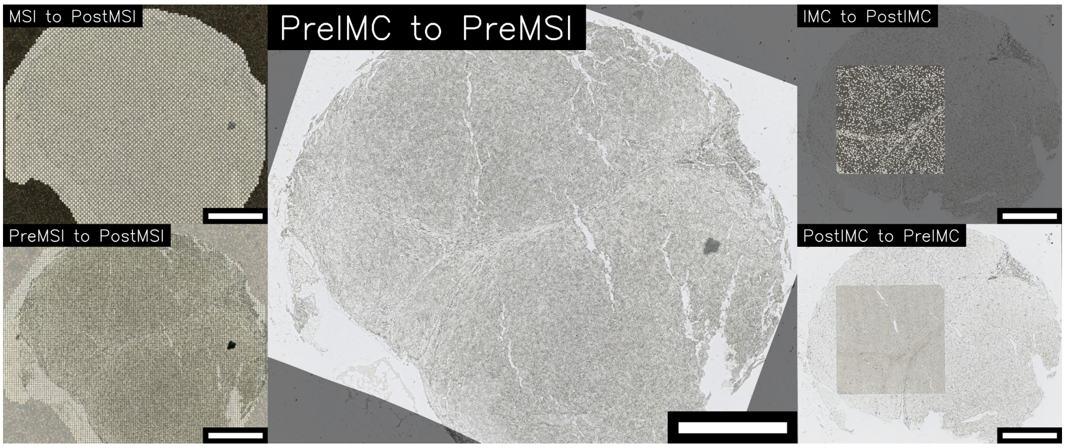

Figure 11: Blended images after co-registration for sample NASH 020.

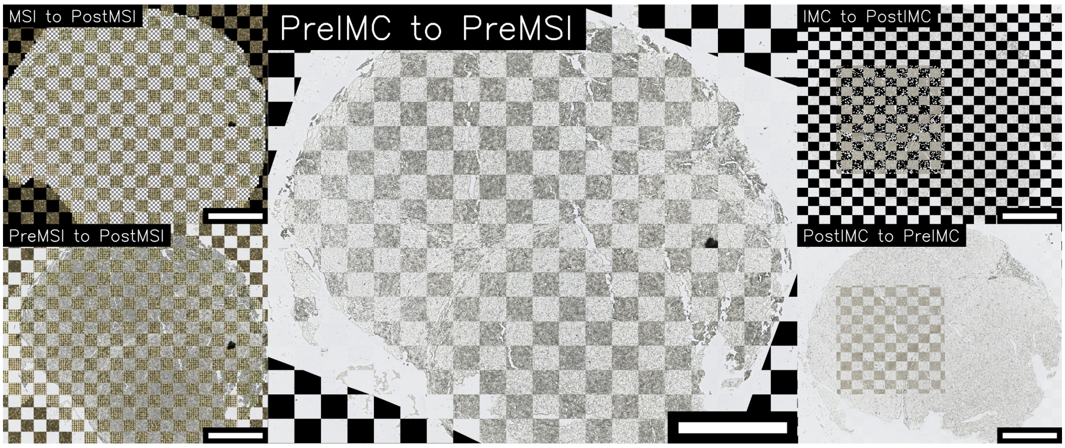

Figure 12: Checkerboard images after co-registration for sample NASH 020.

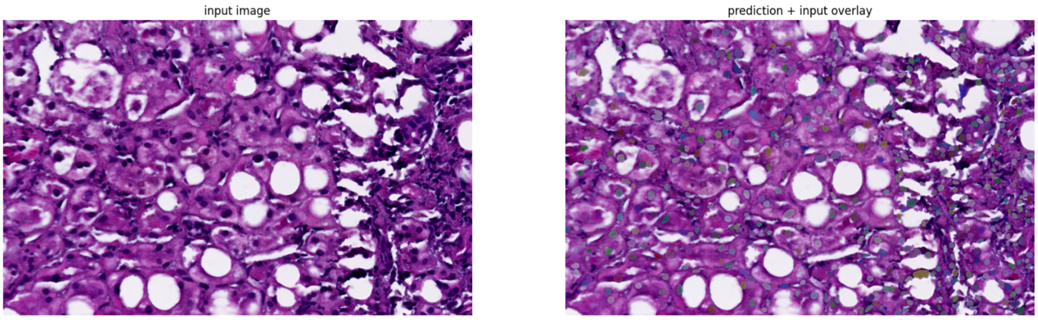

Figure 13: Example image of nuclei detection. Left image: H&E stained image, Right image: overlaid nuclei as detected with StarDist.

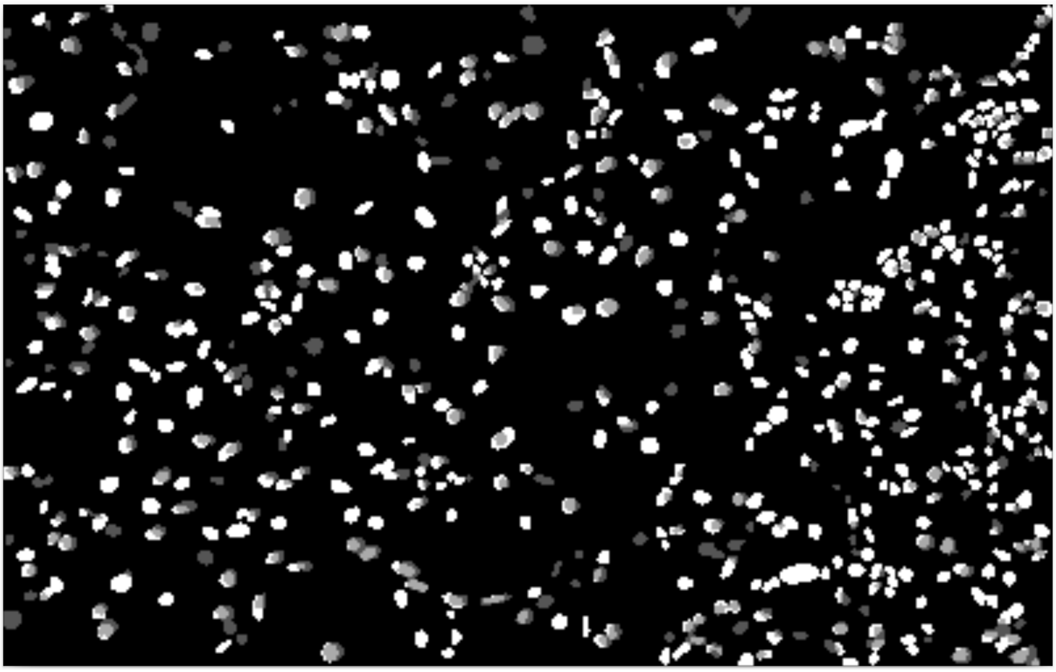

Figure 14: Example image of nuclei detection. White: nuclei from H&E image, Dark gray: nuclei from IMC, Light gray: overlap of the two masks, Black: no nuclei for either modality.

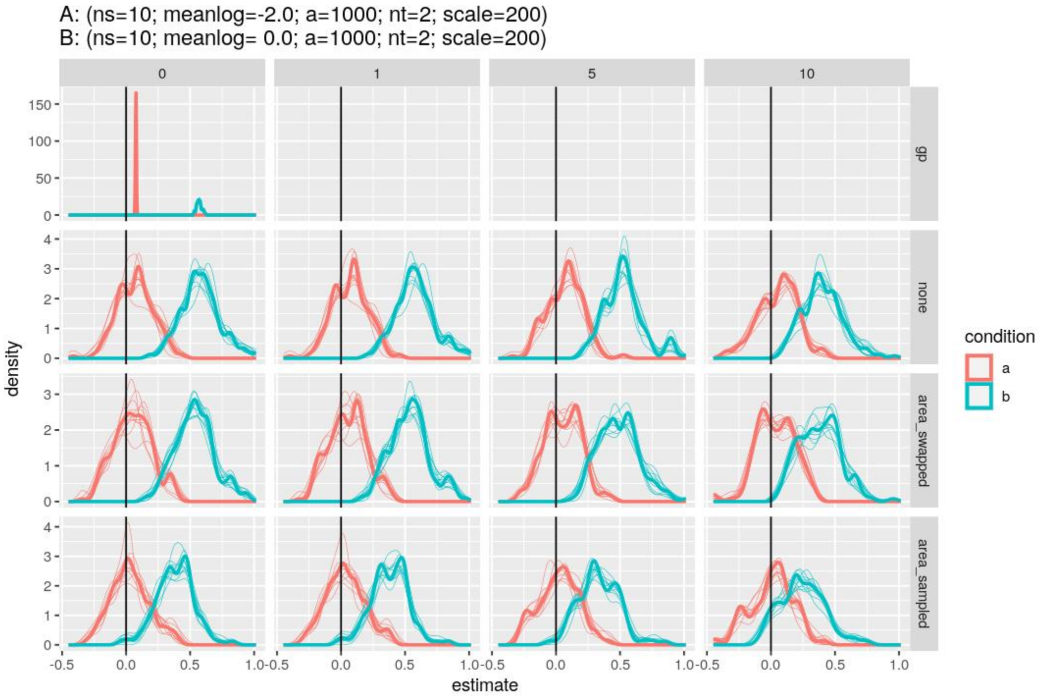

Figure 15: Distribution of coefficients from layer 1 for a simulation with two cell types with MSI stepsize of 30 and MSI pixelsize of 24. Columns: Translation in um. Rows: Area error terms from top to bottom: no error in MSI (ground truth), no error in area (ground truth), shuffled area (areas are similar to ground truth), sampled area (random area).

response	area_error	translation	mean (Intercept)	sd (Intercept)	(Intercept)	mean conditionb	sd conditionb	conditionb
area_a	noerror	0	0.07660	0.000938		0.494000	0.00677	
area_a	none	0	0.07000	0.048700		0.509000	0.06620	
area_a	none	1	0.07110	0.048200		0.507000	0.06650	
area_a	none	5	0.07170	0.047500		0.455000	0.06910	
area_a	none	10	0.05330	0.050500		0.351000	0.07030	
area_a	area_swapped	0	0.05270	0.050100		0.483000	0.06870	
area_a	area_swapped	1	0.05320	0.049300		0.482000	0.06910	
area_a	area_swapped	5	0.05130	0.046800		0.438000	0.07050	
area_a	area_swapped	10	0.03700	0.046200		0.343000	0.06760	
area_a	area_sampled	0	0.03280	0.046000		0.351000	0.06090	
area_a	area_sampled	1	0.03270	0.046100		0.348000	0.06190	
area_a	area_sampled	5	0.02550	0.047400		0.310000	0.06670	
area_a	area_sampled	10	0.00821	0.049300		0.237000	0.06980	
area_b	noerror	0	0.07650	0.000578		0.000202	0.00130	
area_b	none	0	0.09390	0.028100		-0.017800	0.03740	
area_b	none	1	0.09360	0.028100		-0.017600	0.03820	
area_b	none	5	0.08830	0.026500		-0.022200	0.03840	
area_b	none	10	0.07050	0.022600		-0.026900	0.03590	
area_b	area_swapped	0	0.08450	0.025700		-0.024000	0.03740	
area_b	area_swapped	1	0.08450	0.025800		-0.023800	0.03800	
area_b	area_swapped	5	0.08000	0.025500		-0.027800	0.03830	
area_b	area_swapped	10	0.06430	0.022900		-0.032600	0.03690	
area_b	area_sampled	0	0.07210	0.027800		-0.051100	0.03720	
area_b	area_sampled	1	0.07180	0.028000		-0.051600	0.03780	
area_b	area_sampled	5	0.06590	0.026800		-0.054800	0.03740	
area_b	area_sampled	10	0.05010	0.024200		-0.056300	0.03520	

Note:
A: (ns=10; meanlog=-2.0; a=1000; nt=2; scale=200)
B: (ns=10; meanlog= 0.0; a=1000; nt=2; scale=200)

Figure 16: Coefficients of second layer simulation study. First column: cell type. Second column: Error type for cell areas. Third column: translation error. 4th (7th) column: mean of coefficient. 5th (8th) column: standard deviation of coefficient. 6th (9th) column: distribution of coefficient.Rows with $area\_error = noerror$ and $translation = 0$ can be seen as the ground truth.

response	area_error	translation	mean (Intercept)	sd (Intercept)	(Intercept)	mean conditionb	sd conditionb	conditionb
area_a	noerror	0	0.0000	0.0000		0.00e+00	0.00e+00	
area_a	none	0	0.2880	0.3120		8.40e-06	4.29e-05	
area_a	none	1	0.2810	0.3090		6.90e-06	3.51e-05	
area_a	none	5	0.2670	0.2850		2.25e-05	7.63e-05	
area_a	none	10	0.3560	0.3050		8.01e-04	2.56e-03	
area_a	area_swapped	0	0.3740	0.3160		2.27e-05	1.00e-04	
area_a	area_swapped	1	0.3730	0.3190		2.05e-05	9.78e-05	
area_a	area_swapped	5	0.3720	0.3010		5.43e-05	2.27e-04	
area_a	area_swapped	10	0.4170	0.2870		6.98e-04	1.52e-03	
area_a	area_sampled	0	0.4290	0.3040		5.86e-04	1.68e-03	
area_a	area_sampled	1	0.4190	0.2940		5.98e-04	1.65e-03	
area_a	area_sampled	5	0.4440	0.2870		2.26e-03	7.39e-03	
area_a	area_sampled	10	0.5200	0.2990		2.27e-02	6.70e-02	
area_b	noerror	0	0.0000	0.0000		5.33e-01	2.92e-01	
area_b	none	0	0.0260	0.0774		4.65e-01	2.93e-01	
area_b	none	1	0.0262	0.0745		4.63e-01	2.90e-01	
area_b	none	5	0.0239	0.0540		4.38e-01	2.88e-01	
area_b	none	10	0.0385	0.0613		4.15e-01	2.95e-01	
area_b	area_swapped	0	0.0339	0.0806		4.36e-01	2.76e-01	
area_b	area_swapped	1	0.0340	0.0799		4.36e-01	2.77e-01	
area_b	area_swapped	5	0.0380	0.0823		4.27e-01	2.94e-01	
area_b	area_swapped	10	0.0693	0.1060		4.07e-01	3.03e-01	
area_b	area_sampled	0	0.0672	0.1520		2.97e-01	3.11e-01	
area_b	area_sampled	1	0.0686	0.1490		2.97e-01	3.11e-01	
area_b	area_sampled	5	0.0788	0.1320		2.75e-01	3.00e-01	
area_b	area_sampled	10	0.1520	0.1900		2.40e-01	2.80e-01	

Note:

A: (ns=10; meanlog=-2.0; a=1000; nt=2; scale=200)

B: (ns=10; meanlog= 0.0; a=1000; nt=2; scale=200)

Figure 17: P-values of second layer simulation study. First column: cell type. Second column: Error type for cell areas. Third column: translation error. 4th (7th) column: mean of p-values. 5th (8th) column: standard deviation of p-values. 6th (9th) column: distribution of p-values. Rows with $area\_error = noerror$ and $translation = 0$ can be seen as the ground truth.

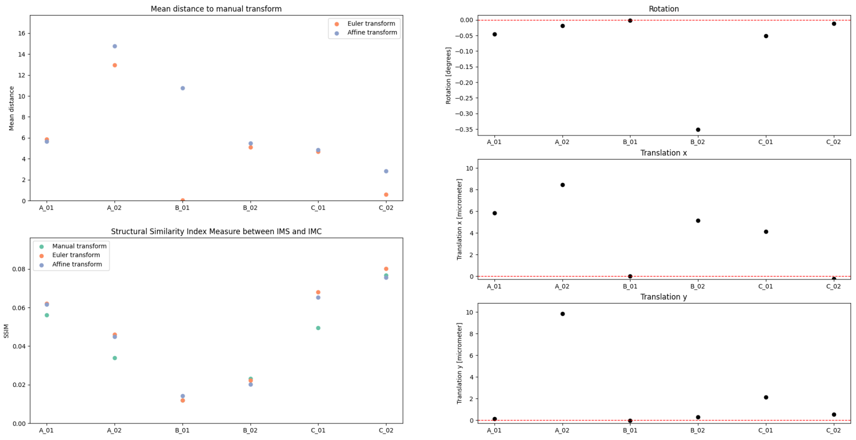

Figure 18: Registration parameters and metrics of MSI to MSI coregistration for the Nunes et al. data. Top left: Mean distance of grid point between the manual and the euler or affine transformation. Bottom left: Structural similarity index measure between IMC and MSI for the different transformations. Right: Euler transformation parameters from top to bottom: rotation, x-translation, y-translation.

**Parameter**	**Value**
**Transfer**	
MALDI Plate Offset	30.0 V
Deflection 1 Delta	70.0 V
Funnel 1 RF	450.0 Vpp
isCID Energy	0.0 eV
Funnel 2 RF	500.0 Vpp
Multipole RF	500.0 Vpp
**Collision Cell**	
Collision Energy	10.0 eV
Collision RF	2900.0 Vpp
**Quadrupole**	
Ion Energy	5.0 eV
Low Mass	m/z 700.00
**Focus Pre TOF**	
Transfer Time	110.0 μs
Pre Pulse Storage	10.0 μs

Table 2: Instrument Parameters

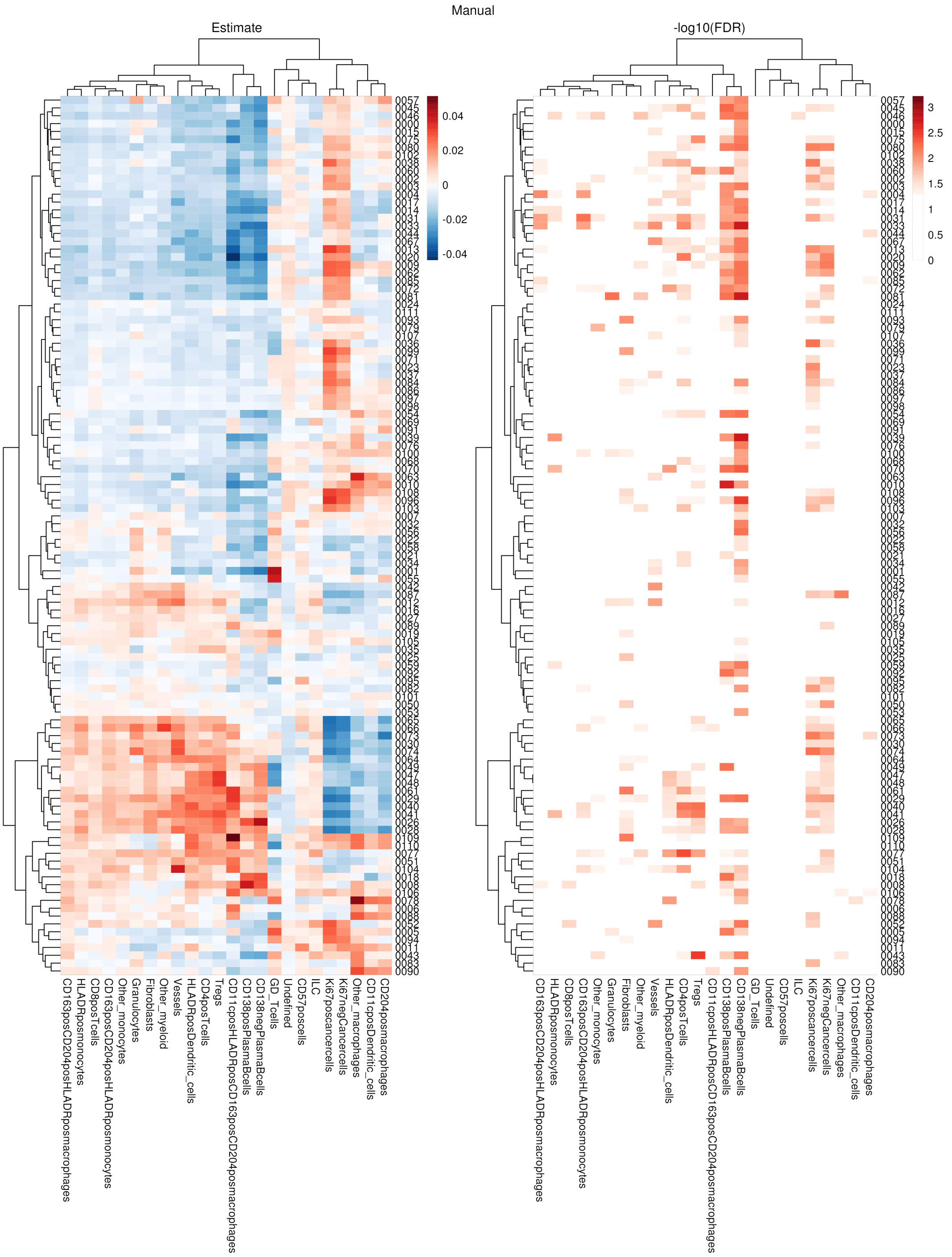

Figure 19: Association results of MSI vs. cell type proportion for the Nunes et al. data. Results for the manual transformation are shown. Left: Heatmap of estimates. Right: Heatmap of -log10(FDR corrected p-values).

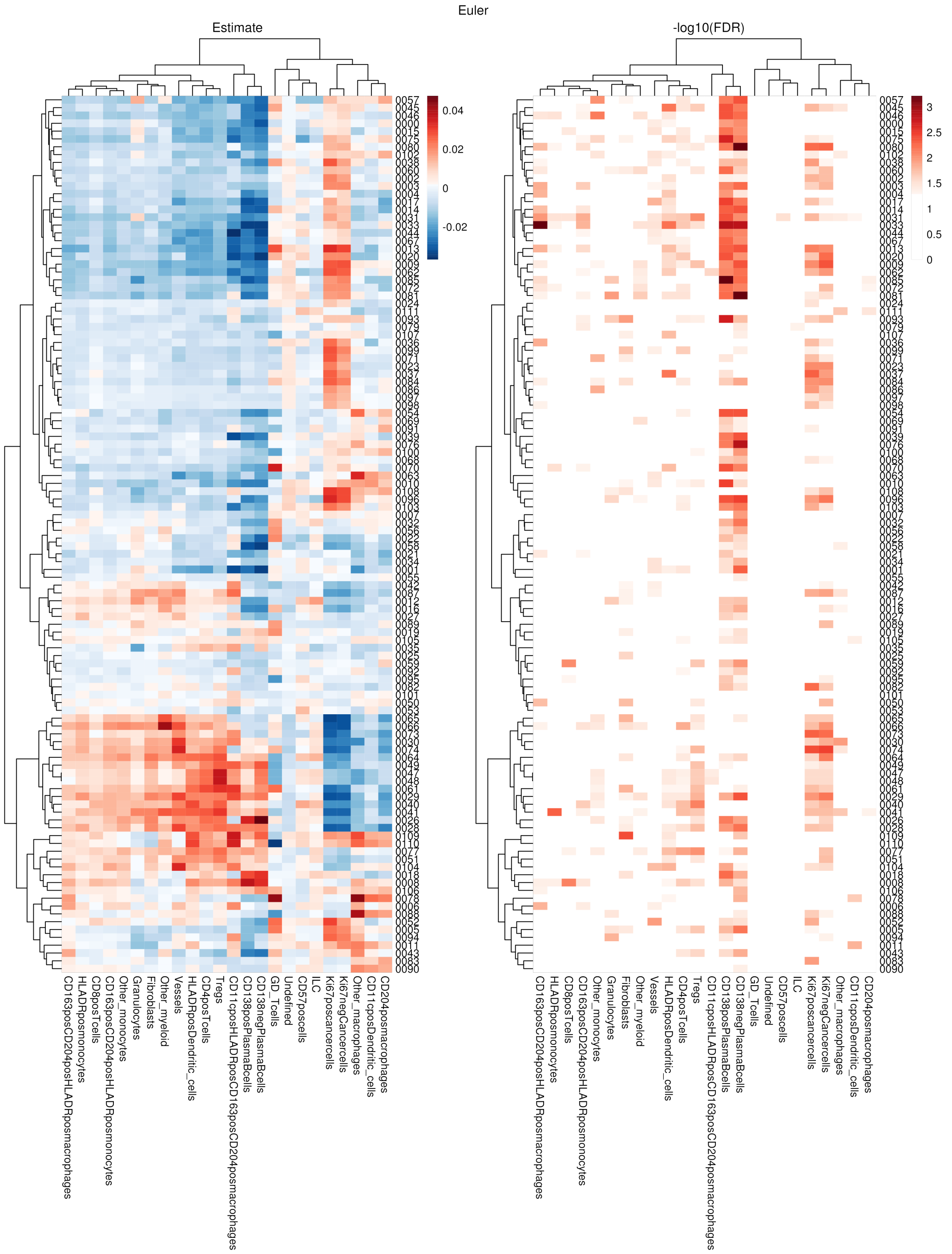

Figure 20: Association results of MSI vs. cell type proportion for the Nunes et al. data. Results for the automatic euler transformation are shown. Left: Heatmap of estimates. Right: Heatmap of -log10(FDR corrected p-values).

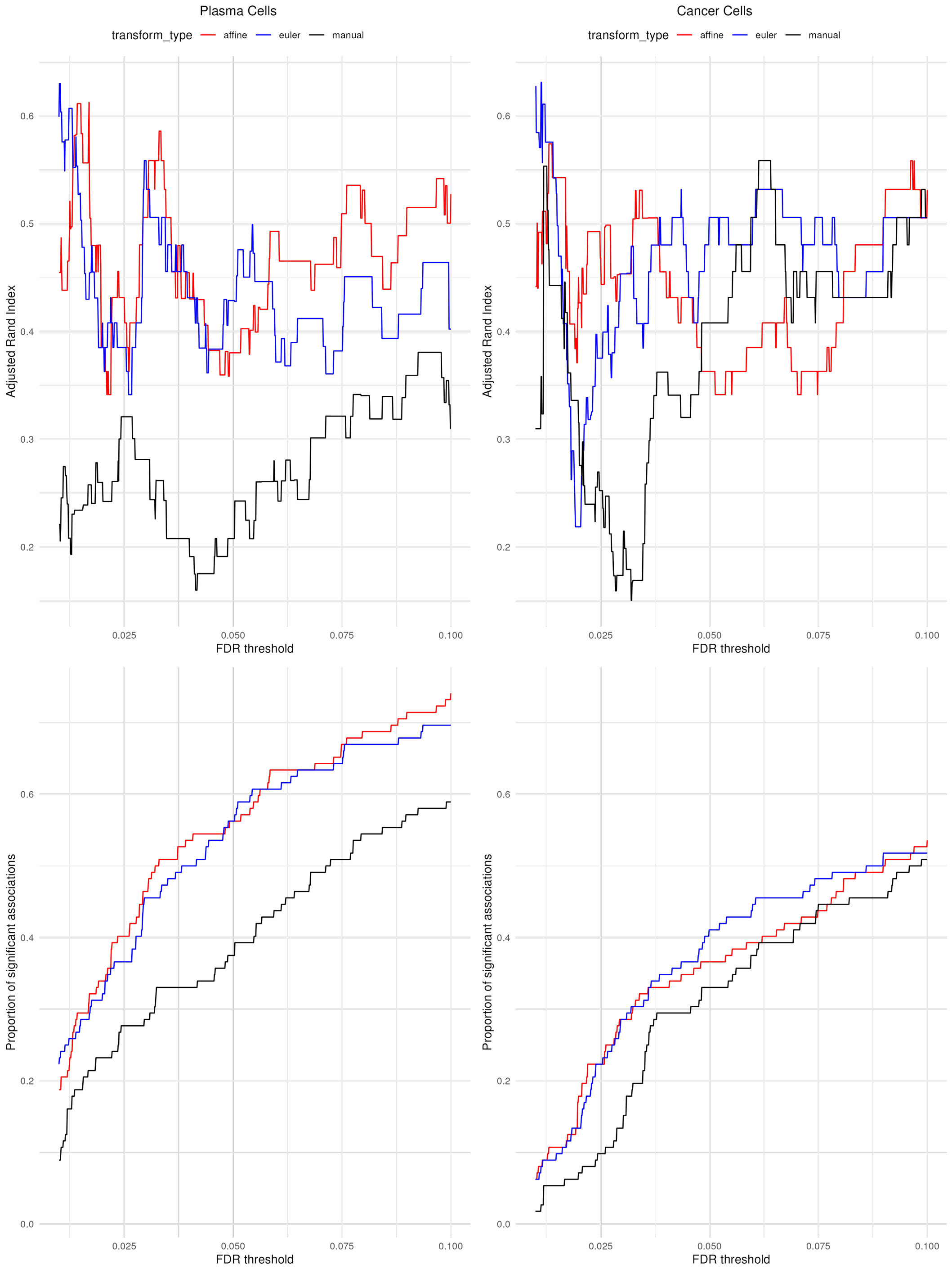

Figure 21: Comparison of modeling results of two celltypes (Plasma Cells and Cancer Cells) that both have two subcelltypes. Top row: Adjusted rand index as a function of FDR threshold, i.e. clustering m/z values into two clusters - significant and not significant - and calculation of the ARI between those two groups. Bottom row: Proportion of significant associations (i.e. both subcelltypes are significant) as a function of FDR threshold. Left column: Plasma Cells. Right column: Cancer Cells.

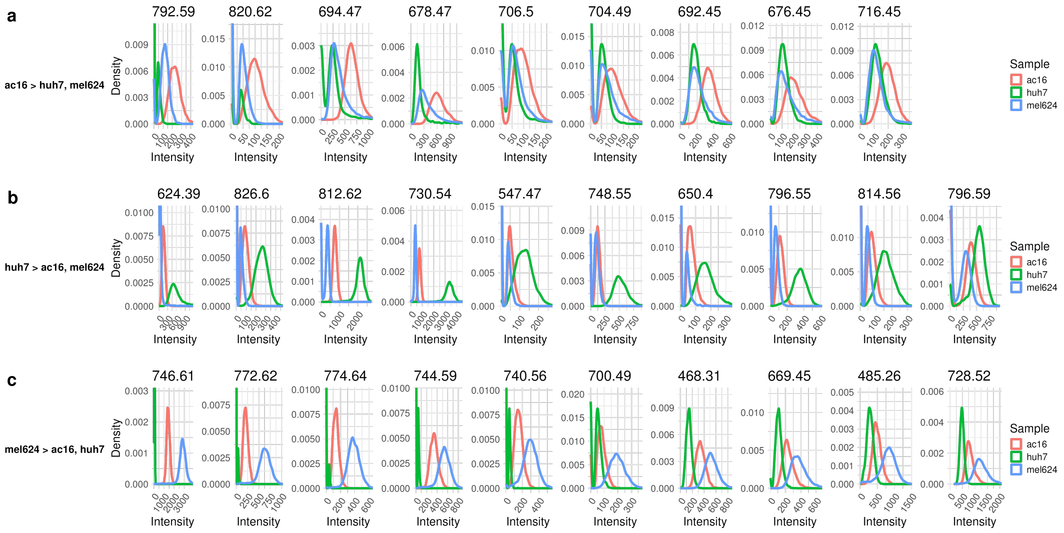

Figure 22: Distribution of peaks with different intensity between in-isolation samples. a) m/z peaks with higher intensity in cell line AC16 compared to the other two cell lines. b) higher in Huh7. c) higher in Mel624. For Huh7 only the top 10 peaks are shown.

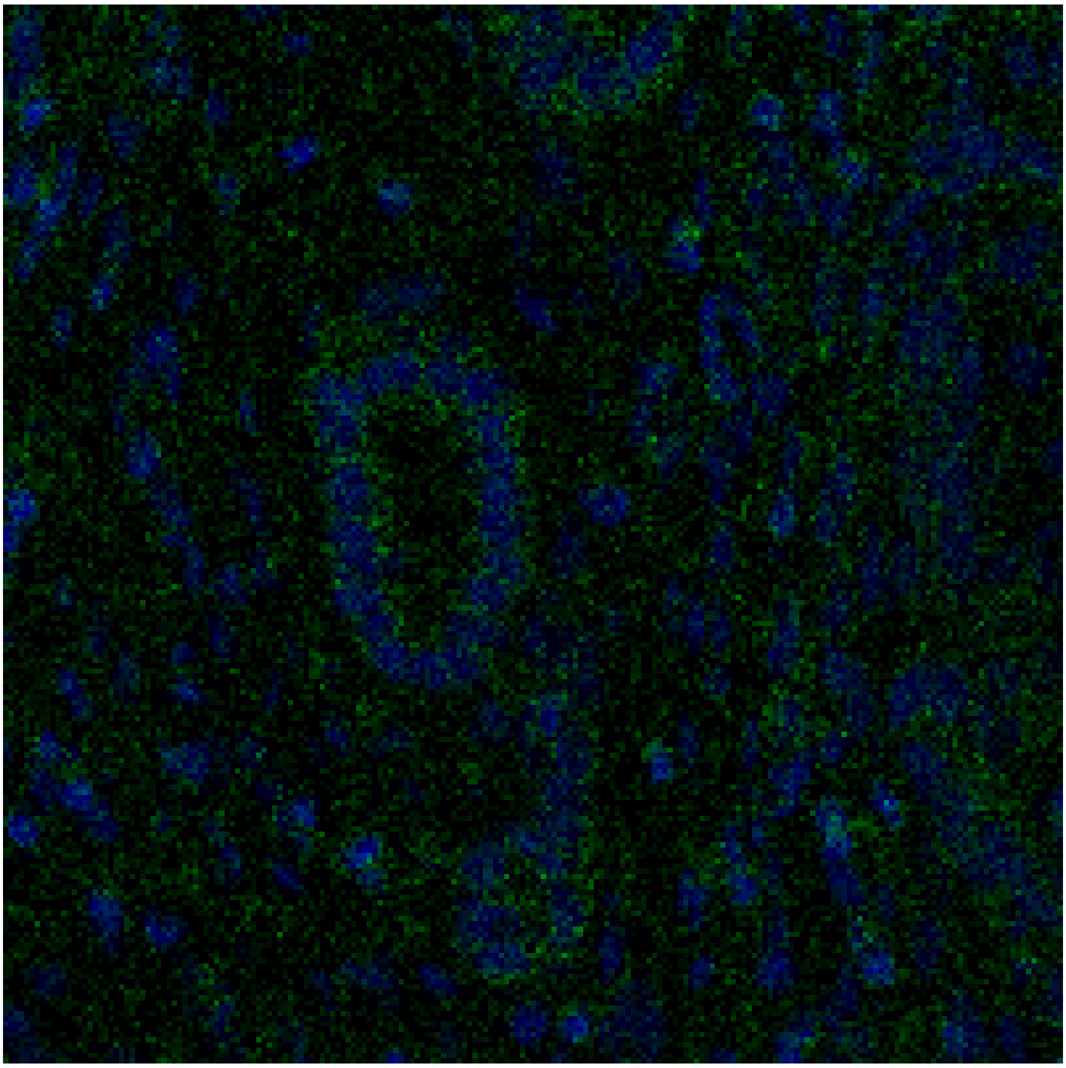

Figure 23: Example IMC image for the Liver TMA. Blue: DNA channel, green: segmentation channel.

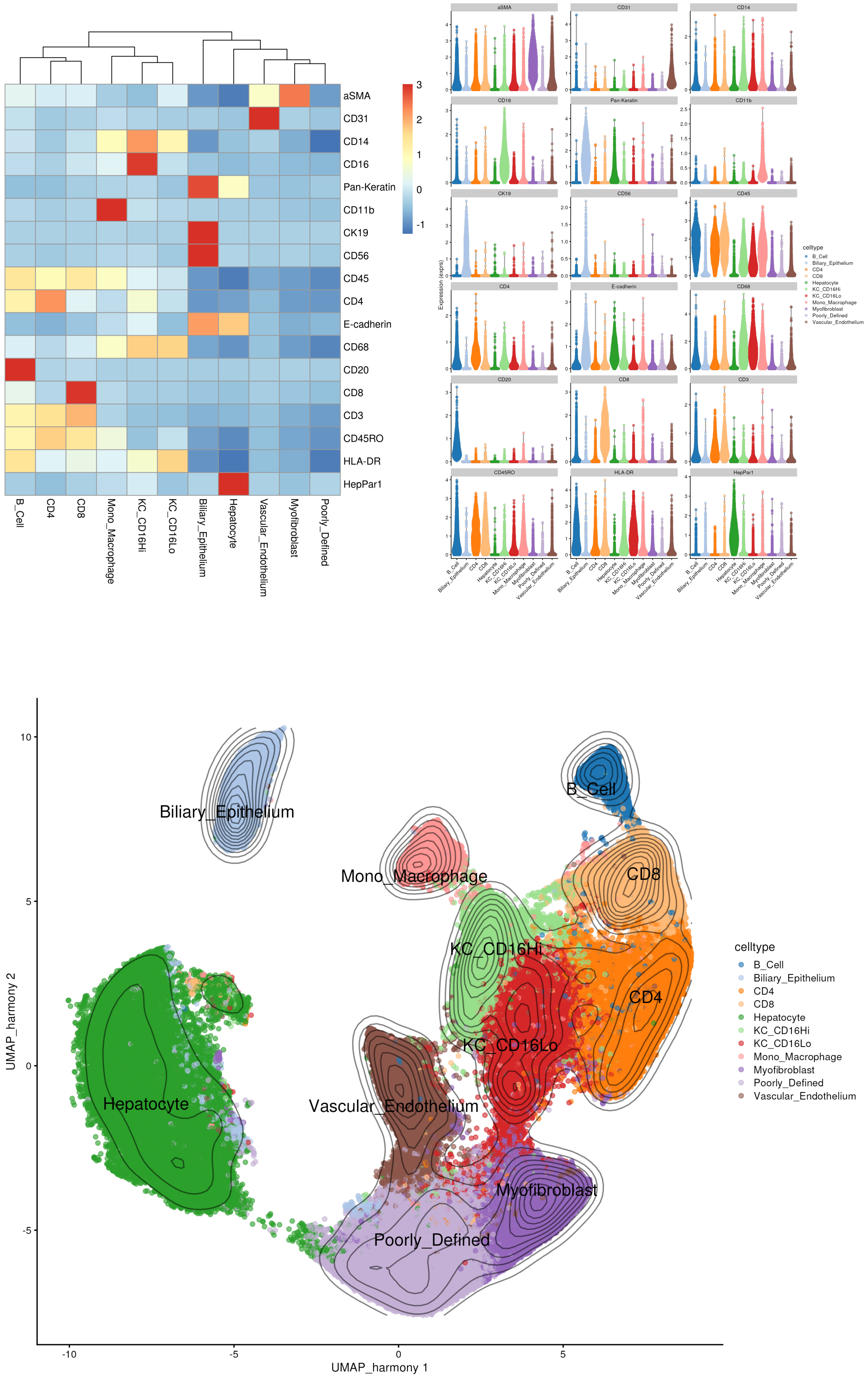

Figure 24: IMC data of Liver tissue. a) Heatmap of row-scaled mean expression per cell type. b) Violin plot of distribution of markers per cell type. c) UMAP of integrated data colored by cell type.

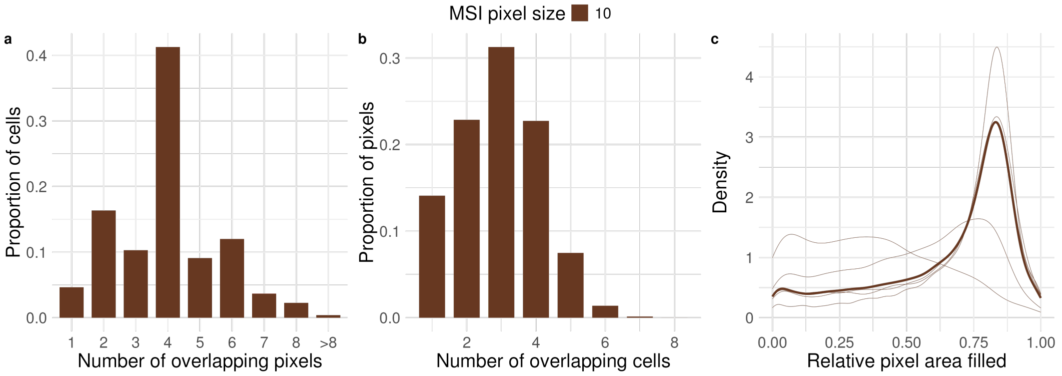

Figure 25: Distributions of overlaps between cells and MSI pixels for the artificial tissue. a) Distribution of number of pixels a cell overlaps with. b) Distribution of number of cells a MSI pixel overlaps with. c) Distribution of relative area filled by cells per MSI pixel.

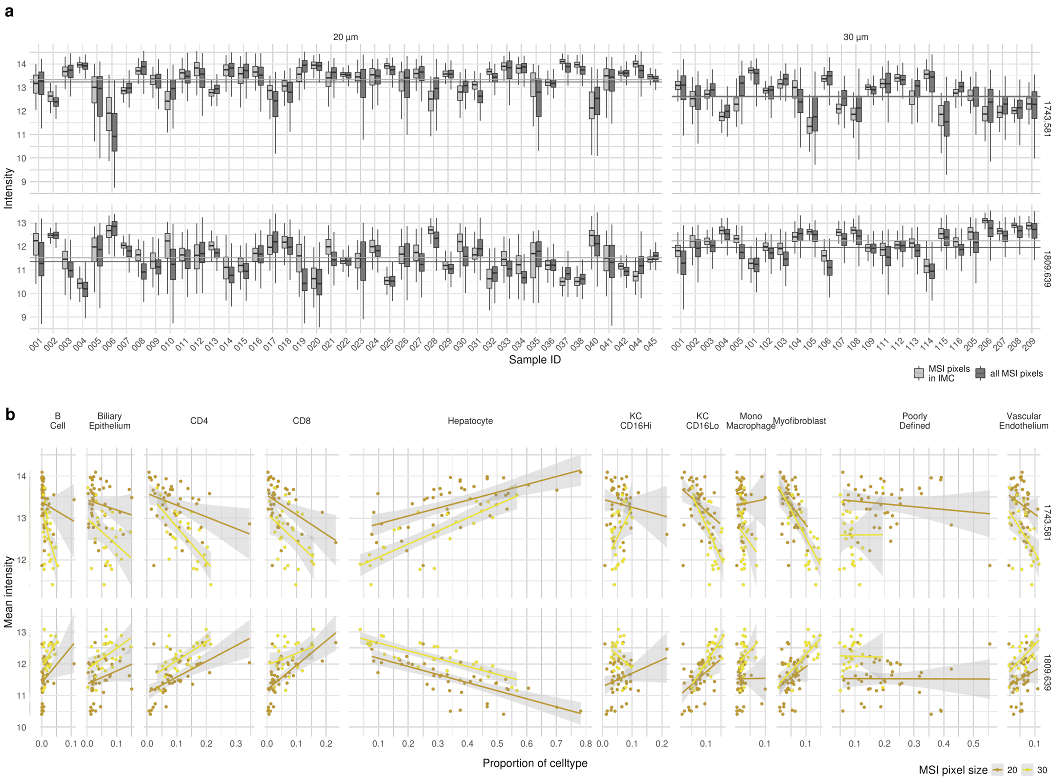

Figure 26: a) Distribution of two example m/z values (rows) for all samples (columns) colored by if all MSI pixels are used (dark gray) or if only MSI pixels on the IMC location are used (light gray). Panel titles are the MSI step size (20 or 30 microns). b) For the same two example m/z values the mean intensity vs. the cell type proportion. Line are the linear regression fit per MSI step size.

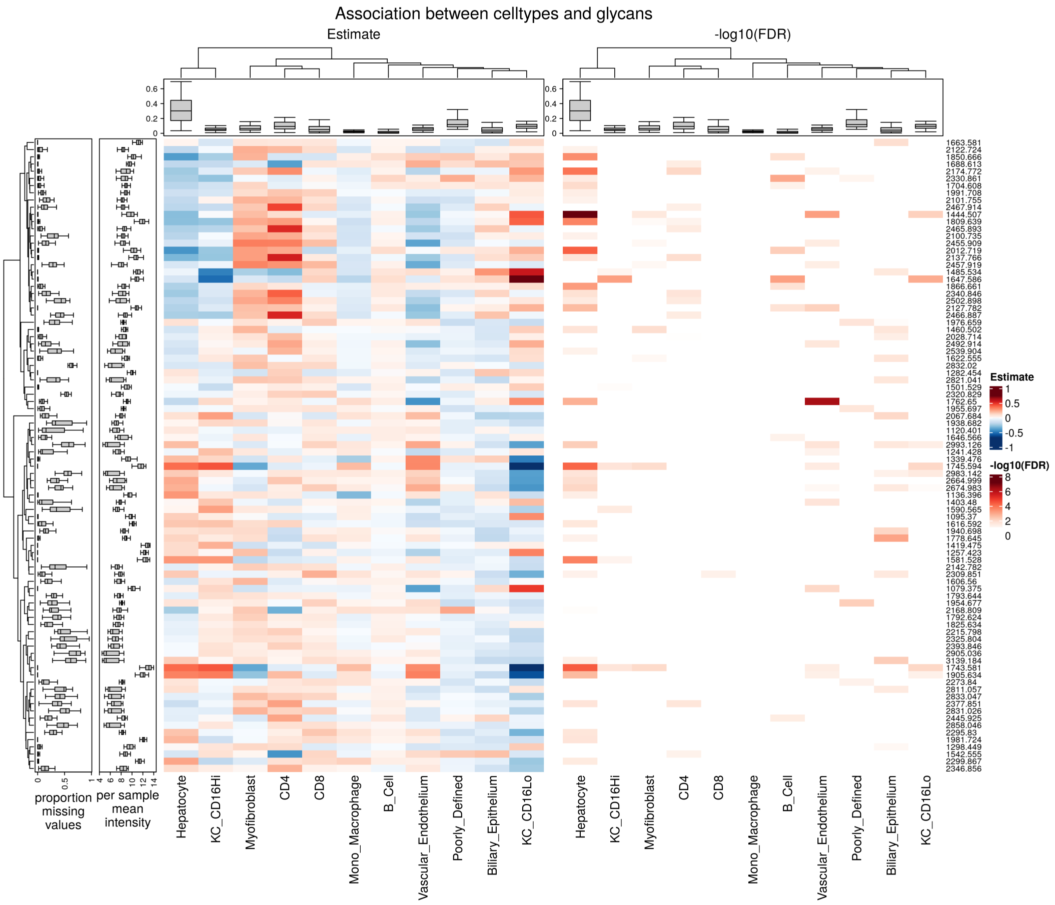

Figure 27: Results of statistical analysis, aggregated per sample. Heatmap per m/z and cell type for effect estimates (a) and -log10 FDR corrected p-values (b).

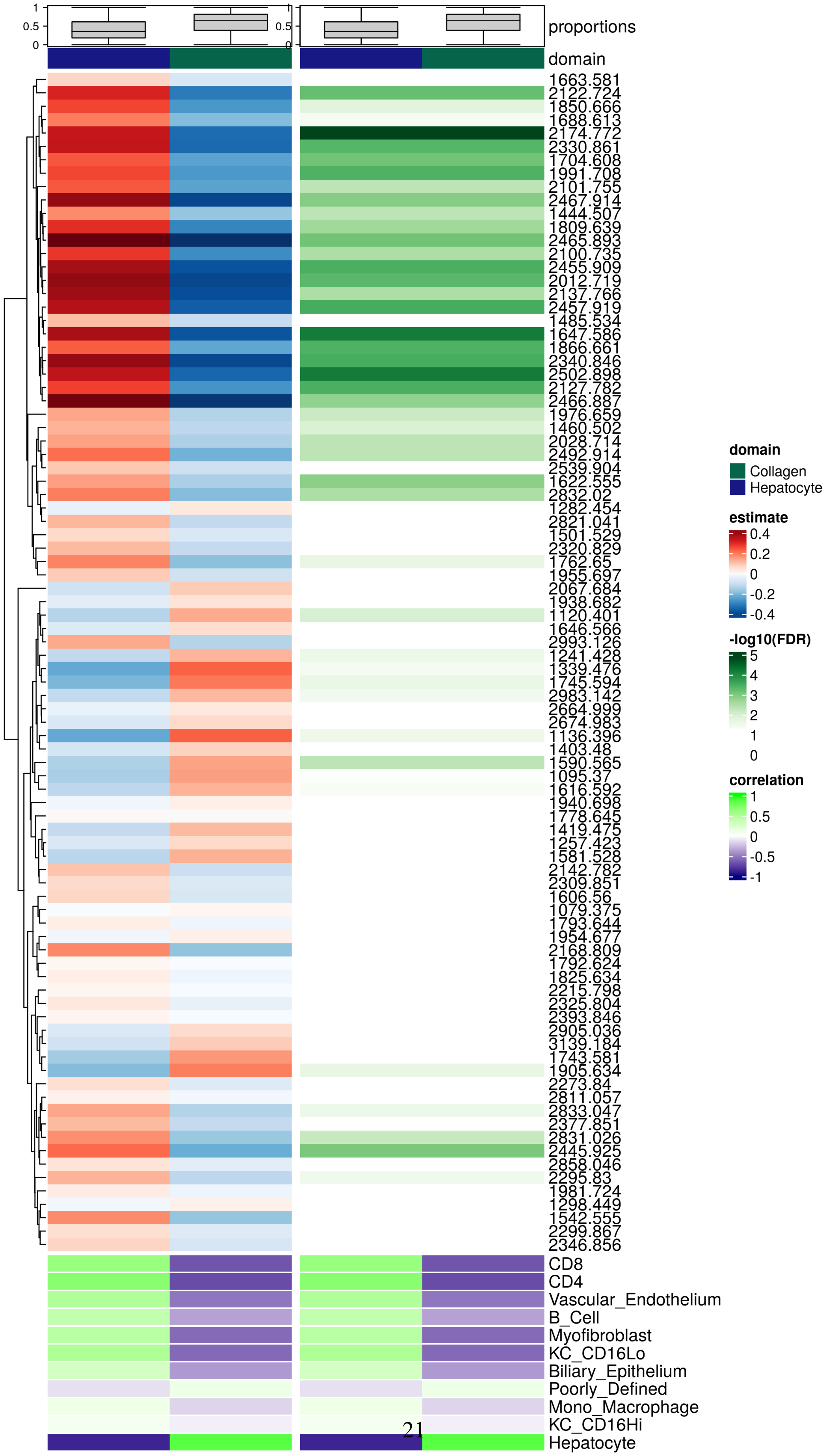

Figure 28: Results of statistical analysis, aggregated per domain and sample. Heatmap per m/z and domain for effect estimates (left) and -log10 FDR corrected p-values (right). Bottom Heatmap shows the correlation of cell type proportion with domain proportion.

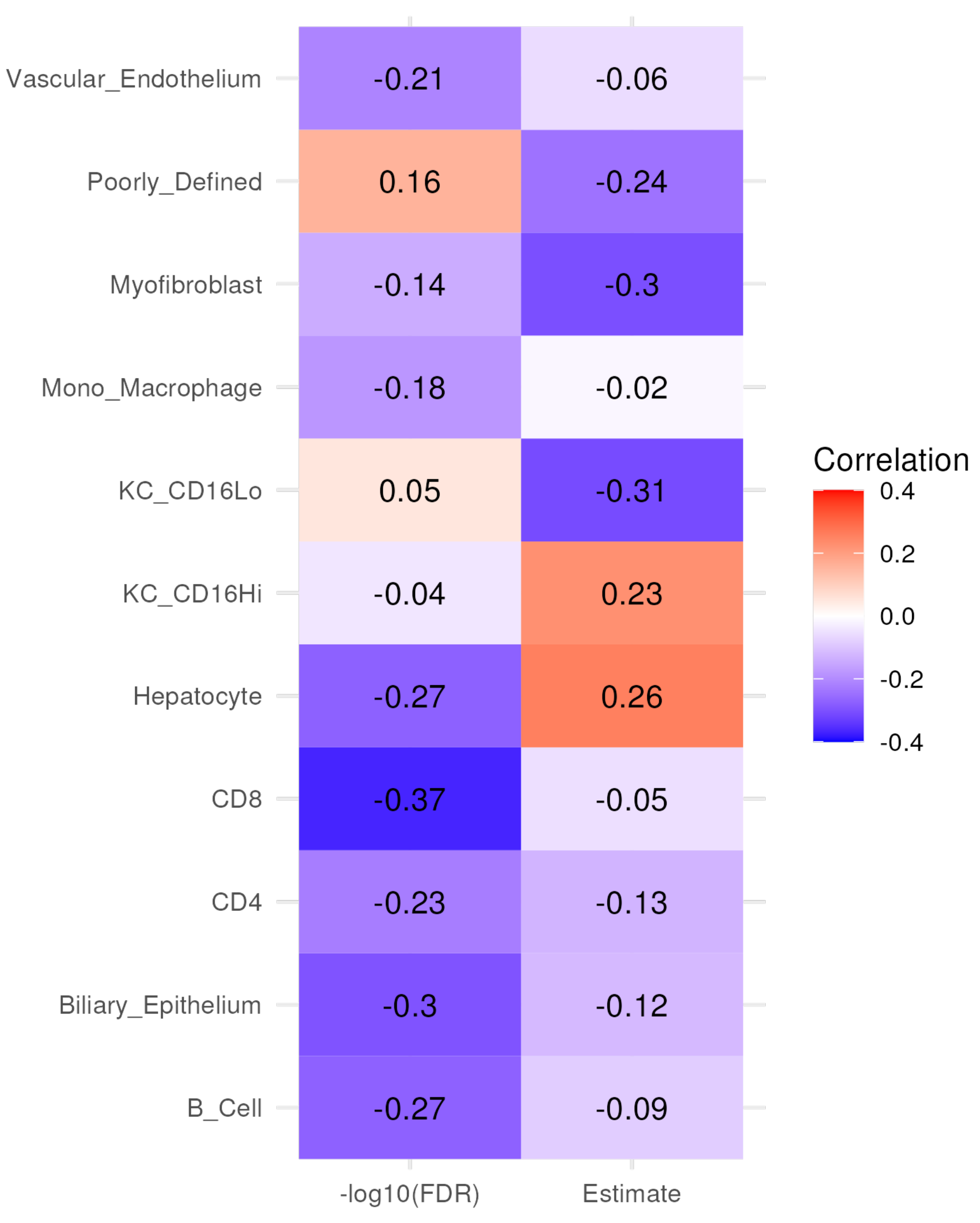

Figure 29: Heatmap of Correlations between either Estimates or p-values per cell type with proportion of missing m/z values.

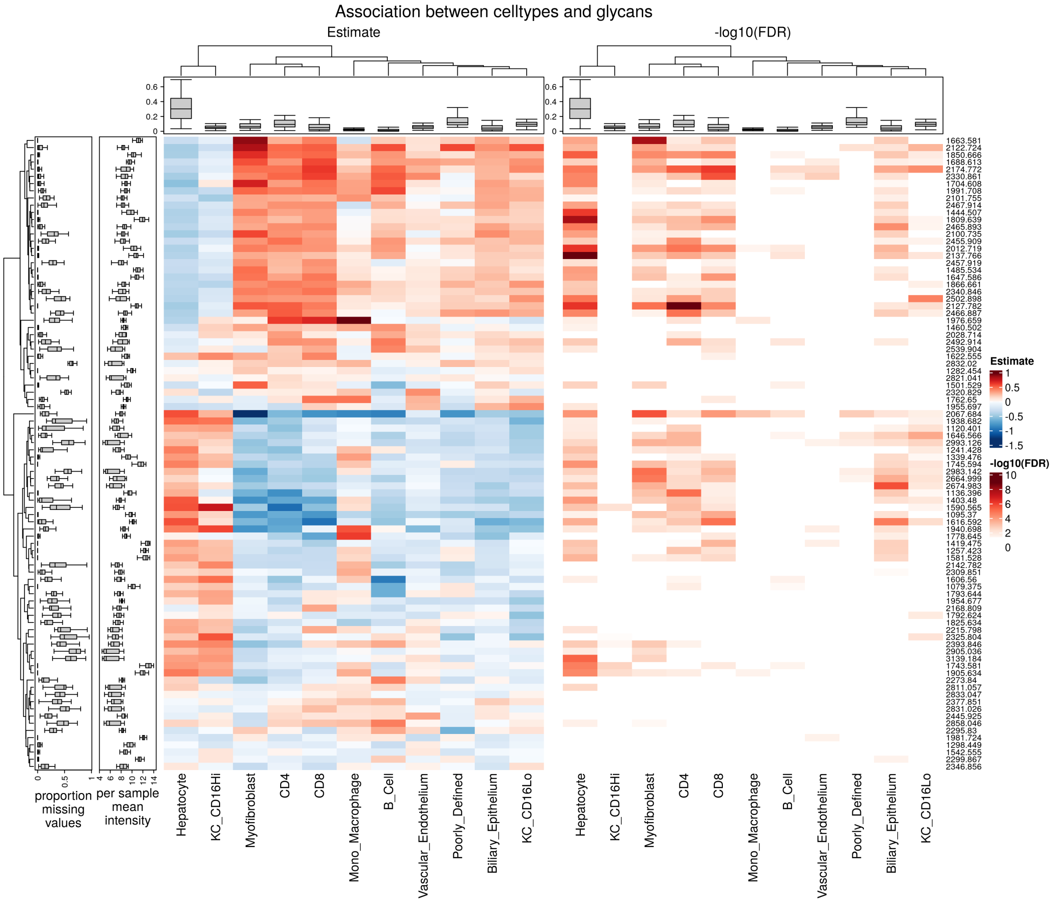

Figure 30: Results of statistical analysis. Heatmap per m/z and cell type for effect estimates (a) and -log10 FDR corrected p-values (b).
