# Supplementary material for: MIMIC: a flexible pipeline to register and summarize IMC-MSI experiments
Source: bioRxiv. 2025 Jul 11:2025.07.08.663623. Preprint. [Version 1] doi: 10.1101/2025.07.08.663623 (PMC12265742; doi:10.1101/2025.07.08.663623)
Supplement: Supplementary file 2 [file media-2.pdf]

MIMIC: a flexible pipeline to register and summarize IMC-MSI experiments

Supplementary Note

Reto Gerber[1†], Jake Griner[2†], Silvia Guglietta[2], Carsten Krieg[3], Mark D. Robinson[1*]

[1]Department of Molecular Life Sciences and SIB Swiss Institute of Bioinformatics, University of Zurich, Zurich, Switzerland.

[2]Department of Regenerative Medicine and Cell Biology, Medical University of South Carolina, Charleston, South Carolina, USA.

[3]Department of Pathology and Laboratory Medicine, Medical University of South Carolina, Charleston, South Carolina, USA.

*Corresponding author(s). E-mail(s): mark.robinson@mls.uzh.ch;
†These authors contributed equally to this work.

1 Simulation Study

The overview of the Simulation is given in Supplementary Figure 1. The simulated images have a size of 1000um x 1000um in which a multi type point pattern with simple sequential inhibition (inhibition distance = 0.975 quantile sqrt(area/pi) of all observed cell areas) is sampled. The intensity is inhomogeneous based on a sampled gaussian random field (GRF, scaled from 0 to 1) where the number of cells is fixed for each simulation condition. Multiple celltypes are simulated, the first has intensity according to the simulated scaled GRF while all others have intensity inverse to the GRF. Cell areas are simulated from a negative binomial with the parameters obtained from fitting on the real cell areas. The distribution of m/z values can be described with a log-normal distribution. Therefore to model the m/z value of a cell (per μm squared) a log-normal distribution is chosen (parameters manually specified). Those parameters can be adjusted per celltype and are the main parameters to evaluate the sensitivity and power.

Next a grid is overlaid representing the MSI pixels. The stepsize is the same as in the experimental data which is 30 μm with a pixelsize of 24 μm. Other parameters are stepsize of 20 and 10, and pixelsize of 30,24,20,10,8. To investigate how important exact registration is a translation is applied in the x-axis of either,

0, 1, 5 or 10 μm. Furthermore the effect of imprecise cell areas are modeled. First by randomly reshuffling of the cell areas (extreme case) or by a rank based reordering (split the areas into multiple subsets based on quantiles, within each subset invert order of ranks of areas). This second approach tries to mimic the fact that IMC and MSI were not obtained from the same tissue slice but from adjacent slices, meaning the cell areas will not be exactly the same. Randomly adding or removing cells would have been another option which wasn't used here. For those last three parameters the estimates from model fitting can be directly compared to each other since they rely on the same underlying data. Next, three different error terms are added to the aggregated MSI image. The first is an error based on the neighbouring pixels, essentially smoothing the signal. The second is an uncorrelated error sampled from a log-normal. The third is correlated error drawn from a GRF scaled between 0 and a max value (of 500). Additionally the cell type areas per pixel are aggregated as well leading to the final structure of the observed data.

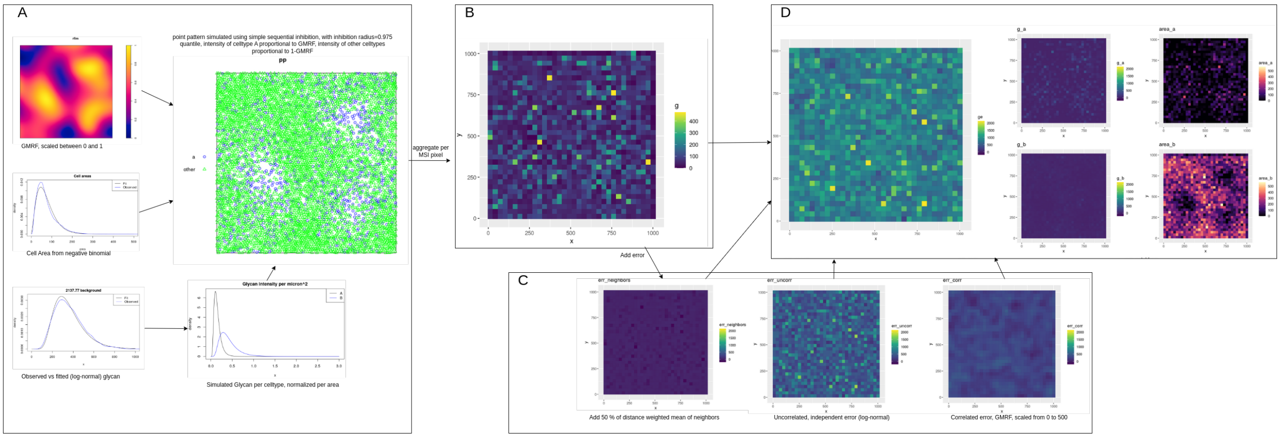

Fig. 1 Overview of the Simulation Framework. A) Top-left image: Gaussian Random Field for intensity for point process. Middle-left graph: Distribution of observed cell areas and fitted Negative Binomial. Bottom-left graph: Distribution of MSI intensities and fitted log-normal. Bottom-middle: Distribution of used MSI intensity normalized by unit area per cell type. Top-right image: Sampled point pattern (using simple sequential inhibition), color is the cell type. B) Aggregated MSI intensities. C) Error-terms added to image in B). Left: Error based on intensity of neighbouring pixels. Middle: Uncorrelated error. Right: Correlated error, sampled from Gaussian Markov Random Field. D) Left image: Final observed image, sum of B) and C). Right images: Observed areas per pixel and cell type. Middle images: Actual signal per cell type.

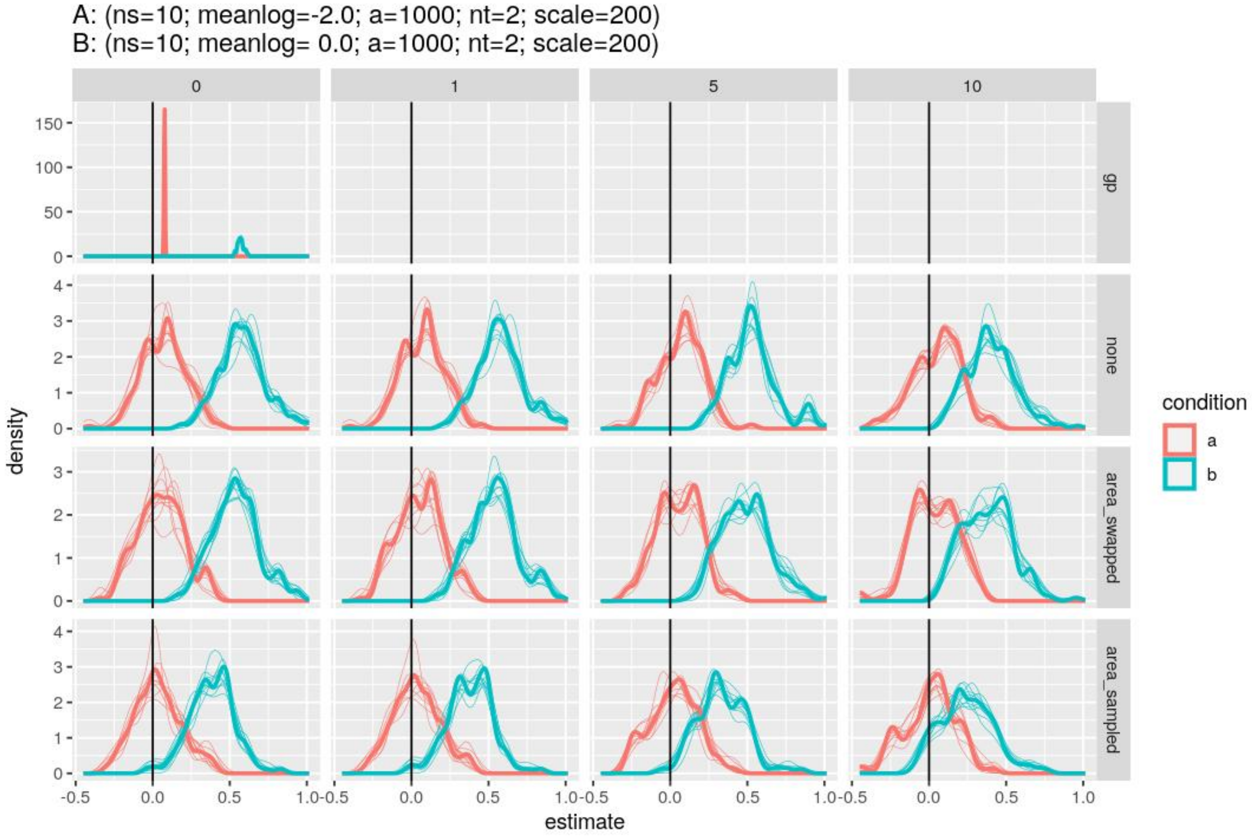

Fig. 2 Distribution of coefficients from layer 1 for a simulation with two cell types with MSI stepsize of 30 and MSI pixelsize of 24. Columns: Translation in um. Rows: Area error terms from top to bottom: no error in MSI (ground truth), no error in area (ground truth), shuffled area (areas are similar to ground truth), sampled area (random area).

response	area_error	translation	mean (Intercept)	sd (Intercept)	(Intercept)	mean conditionb	sd conditionb	conditionb
area_a	noerror	0	0.07660	0.000938		0.494000	0.00677	
area_a	none	0	0.07000	0.048700		0.509000	0.06620	
area_a	none	1	0.07110	0.048200		0.507000	0.06650	
area_a	none	5	0.07170	0.047500		0.455000	0.06910	
area_a	none	10	0.05330	0.050500		0.351000	0.07030	
area_a	area_swapped	0	0.05270	0.050100		0.483000	0.06870	
area_a	area_swapped	1	0.05320	0.049300		0.482000	0.06910	
area_a	area_swapped	5	0.05130	0.046800		0.438000	0.07050	
area_a	area_swapped	10	0.03700	0.046200		0.343000	0.06760	
area_a	area_sampled	0	0.03280	0.046000		0.351000	0.06090	
area_a	area_sampled	1	0.03270	0.046100		0.348000	0.06190	
area_a	area_sampled	5	0.02550	0.047400		0.310000	0.06670	
area_a	area_sampled	10	0.00821	0.049300		0.237000	0.06980	
area_b	noerror	0	0.07650	0.000578		0.000202	0.00130	
area_b	none	0	0.09390	0.028100		-0.017800	0.03740	
area_b	none	1	0.09360	0.028100		-0.017600	0.03820	
area_b	none	5	0.08830	0.026500		-0.022200	0.03840	
area_b	none	10	0.07050	0.022600		-0.026900	0.03590	
area_b	area_swapped	0	0.08450	0.025700		-0.024000	0.03740	
area_b	area_swapped	1	0.08450	0.025800		-0.023800	0.03800	
area_b	area_swapped	5	0.08000	0.025500		-0.027800	0.03830	
area_b	area_swapped	10	0.06430	0.022900		-0.032600	0.03690	
area_b	area_sampled	0	0.07210	0.027800		-0.051100	0.03720	
area_b	area_sampled	1	0.07180	0.028000		-0.051600	0.03780	
area_b	area_sampled	5	0.06590	0.026800		-0.054800	0.03740	
area_b	area_sampled	10	0.05010	0.024200		-0.056300	0.03520	

Note:

A: (ns=10; meanlog=-2.0; a=1000; nt=2; scale=200)

B: (ns=10; meanlog= 0.0; a=1000; nt=2; scale=200)

Fig. 3 Coefficients of second layer simulation study. First column: cell type. Second column: Error type for cell areas. Third column: translation error. 4th (7th) column: mean of coefficient. 5th (8th) column: standard deviation of coefficient. 6th (9th) column: distribution of coefficient.Rows with $area\_error = noerror$ and $translation = 0$ can be seen as the ground truth.

response	area_error	translation	mean (Intercept)	sd (Intercept)	(Intercept)	mean conditionb	sd conditionb	conditionb
area_a	noerror	0	0.0000	0.0000		0.00e+00	0.00e+00	
area_a	none	0	0.2880	0.3120		8.40e-06	4.29e-05	
area_a	none	1	0.2810	0.3090		6.90e-06	3.51e-05	
area_a	none	5	0.2670	0.2850		2.25e-05	7.63e-05	
area_a	none	10	0.3560	0.3050		8.01e-04	2.56e-03	
area_a	area_swapped	0	0.3740	0.3160		2.27e-05	1.00e-04	
area_a	area_swapped	1	0.3730	0.3190		2.05e-05	9.78e-05	
area_a	area_swapped	5	0.3720	0.3010		5.43e-05	2.27e-04	
area_a	area_swapped	10	0.4170	0.2870		6.98e-04	1.52e-03	
area_a	area_sampled	0	0.4290	0.3040		5.86e-04	1.68e-03	
area_a	area_sampled	1	0.4190	0.2940		5.98e-04	1.65e-03	
area_a	area_sampled	5	0.4440	0.2870		2.26e-03	7.39e-03	
area_a	area_sampled	10	0.5200	0.2990		2.27e-02	6.70e-02	
area_b	noerror	0	0.0000	0.0000		5.33e-01	2.92e-01	
area_b	none	0	0.0260	0.0774		4.65e-01	2.93e-01	
area_b	none	1	0.0262	0.0745		4.63e-01	2.90e-01	
area_b	none	5	0.0239	0.0540		4.38e-01	2.88e-01	
area_b	none	10	0.0385	0.0613		4.15e-01	2.95e-01	
area_b	area_swapped	0	0.0339	0.0806		4.36e-01	2.76e-01	
area_b	area_swapped	1	0.0340	0.0799		4.36e-01	2.77e-01	
area_b	area_swapped	5	0.0380	0.0823		4.27e-01	2.94e-01	
area_b	area_swapped	10	0.0693	0.1060		4.07e-01	3.03e-01	
area_b	area_sampled	0	0.0672	0.1520		2.97e-01	3.11e-01	
area_b	area_sampled	1	0.0686	0.1490		2.97e-01	3.11e-01	
area_b	area_sampled	5	0.0788	0.1320		2.75e-01	3.00e-01	
area_b	area_sampled	10	0.1520	0.1900		2.40e-01	2.80e-01	

Note:
A: (ns=10; meanlog=-2.0; a=1000; nt=2; scale=200)
B: (ns=10; meanlog= 0.0; a=1000; nt=2; scale=200)

Fig. 4 P-values of second layer simulation study. First column: cell type. Second column: Error type for cell areas. Third column: translation error. 4th (7th) column: mean of p-values. 5th (8th) column: standard deviation of p-values. 6th (9th) column: distribution of p-values. Rows with $area\_error = noerror$ and $translation = 0$ can be seen as the ground truth.
